# FMS-Like Tyrosine Kinase 3 (FLT3) and Nucleophosmin 1 (NPM1) in Iranian Adult Acute Myeloid Leukemia Patients with Normal Karyotypes: Mutation Status and Clinical and Laboratory Characteristics

**DOI:** 10.4274/tjh.2016.0489

**Published:** 2017-12-01

**Authors:** Narges Rezaei, Nargess Arandi, Behnaz Valibeigi, Sezaneh Haghpanah, Mehdi Khansalar, Mani Ramzi

**Affiliations:** 1 Hematology Research Center, Shiraz University of Medical Sciences, Shiraz, Iran; 2 Department of Pathology, Faculty of Medicine, Shiraz University of Medical Sciences, Shiraz, Iran; 3 Namazi Hospital, Shiraz University of Medical Sciences, Shiraz, Iran

**Keywords:** Acute myeloid leukemia, Gene mutation, FLT3, NPM1

## Abstract

**Objective:**

In this study, we evaluated the frequency of FMS-like tyrosine kinase 3 (FLT3-ITD and FLT3-TKD) and nucleophosmin (NPM1) mutations in Iranian patients with cytogenetically normal acute myeloid leukemia (CN-AML). The clinical and laboratory characteristics were compared between wild-type and mutant cases.

**Materials and Methods:**

Seventy newly diagnosed de novo AML patients were recruited at the time of diagnosis prior to chemotherapy; among them, 54 had CN-AML. For detecting mutations, the FLT3 and NPM1 genes were amplified by the polymerase chain reaction method, followed by direct sequencing.

**Results:**

Our results showed that the frequencies of FLT3-ITD, FLT3-TKD, and NPM1 mutations in CN-AML patients were 25.9%, 5.9%, and 20.8%, respectively. The most frequent NPM1 mutation type was the type A mutation. The FLT3-ITD mutation was seen more frequently in non-M3 patients compared with M3 patients. No mutation was observed in either the FLT3-TKD or the NPM1 gene in patients in the M3 French-American-British group. There was no significant association between the presence of FLT3-ITD and NPM1 mutations in CN-AML patients (p>0.05). The frequency of FLT3-ITD, FLT3-TKD, and NPM1 mutation was higher in CN-AML patients in comparison with AML patients with cytogenetic aberrations, although the differences were not statistically significant (p>0.05). There were no significant differences in mean white blood cell and platelet counts, serum hemoglobin levels, and bone marrow blast percentages between patients with wild-type and mutant FLT3-ITD and NPM1 genes (p>0.05). No difference was observed in the frequency of FLT3-ITD or NPM1 mutation regarding age or sex (p>0.05).

**Conclusion:**

Given the high stability of NPM1 during the disease course, it can be used in combination with FLT3 as well as other known genetic markers to monitor patients, especially for minimal residual disease detection.

## Abstract (tr)

**Amaç:**

Bu çalışmada, İran’lı normal sitogenetikli akut miyeloid lösemi (NS-AML) hastalarında FMS-benzeri tirozin kinaz 3 (FLT3-ITD ve FLT3-TKD) ile nükleofosmin 1 (NPM1) mutasyonlarının sıklığını değerlendirdik. Mutant olmayan (yabanıl-wild-type) ve mutant olgular klinik ve laboratuvar özellikler açısından mukayese edildi.

**Gereç ve Yöntemler:**

Yetmiş yeni tanı de novo AML hastası kemoterapi uygulanması öncesinde çalısmaya dahil edildi; bunların 54’ü NS-AML idi. Mutasyonları tespit etmek için, FLT3 ve NPM1 genleri polimeraz zincir reaksiyonu ile amplifiye edildi ve bu işlemi direkt dizileme takip etti.

**Bulgular:**

NS-AML hastalarında FLT3-ITD, FLT3-TKD ve NPM1 mutasyonlarının sıklıkları sırasıyla %25,9; %5,9 ve %20,8 olarak bulunmuştur. En sık gözlenen NPM1 mutasyon tipi, tip A mutasyonuydu. FLT3-ITD mutasyonu M3 hastalarına göre M3-dışı olgularda daha sık görülmekteydi. Fransız-Amerikan-İngiliz M3 grubundaki hastalarda FLT3-TKD veya NPM1 genine ait mutasyon tespit edilmedi. NS-AML hastalarında FLT3-ITD ve NPM1 mutasyonlarının varlığı açısından anlamlı ilişki yoktu (p>0,05). FLT3-ITD, FLT3-TKD ve NPM1 mutasyon sıklığı, her ne kadar istatistiksel olarak anlamlı farklılık saptanmasa da (p>0,05), NS-AML hastalarında sitogenetik aberasyonu olan AML olgularına göre daha fazlaydı. FLT3-ITD ve NPM1 genleri açısından mutant olan ve olmayan hastalarda ortalama lökosit ve trombosit sayıları, serum hemoglobin düzeyleri ve kemik iliği blast yüzdeleri arasında anlamlı farklılık yoktu (p>0,05). Yaş ve cinsiyete göre FLT3-ITD veya NPM1 mutasyonlarının sıklıkları açısından farklılık tespit edilmedi (p>0,05).

**Sonuç:**

NPM1 hastalık sürecindeki yüksek kararlılığı nedeniyle, özellikle minimal kalıntı hastalık tespiti açısından FLT3 veya diğer bilinen genetik belirteçler ile kombine olarak hastaların izlenmesinde kullanılabilir.

## INTRODUCTION

Acute myeloid leukemia (AML) is the most common hematologic malignancy, characterized by uncontrolled proliferation of hematopoietic stem cells resulting in abnormal accumulation of myeloblasts [1]. Generally, based on the cytogenetic abnormalities, the prognosis of AML patients is categorized into three risk groups: good, intermediate, and poor [2]. However, about 50% of AML patients have the normal cytogenetic feature (CN-AML), which represents a diverse subset of patients who are usually classified into an intermediate risk group [3]. Recently, assessment of molecular abnormalities has proven to be a useful marker for risk stratification of these patients into good and poor risk subgroups [3,4,5,6]. In this regard, somatic mutations of the FMS-like tyrosine kinase 3 (FLT3), nucleophosmin 1 (NPM1), and Wilms’ tumor 1 (WT1) genes have been well studied [3,7,8,9].

FLT3 is a member of the class III receptor tyrosine kinase (RTK) family, normally expressed in early bone marrow precursors and playing an important role in the regulation of hematopoietic cell proliferation and differentiation [10]. Binding of the FLT3 ligand to its receptor recruits and activates several signaling molecules affecting cell proliferation, differentiation, and survival [11]. The FLT3 receptor consists of five extracellular immunoglobulin-like domains (Ig1-Ig5), a transmembrane domain, a juxtamembrane domain (JM), and the two intracellular tyrosine kinase domains (TK1 and TK2) [12,13,14]. FLT3 is one of the most frequently mutated genes as approximately 30% of all AML patients have a mutated form of it [15]. Two types of activating mutations have been identified in the FLT3 gene: internal tandem duplication (FLT3-ITD) of the region between exon 11 and 12 in the JM domain (occurring in 20%-25% of AML patients), and a point mutation at codon 835 of exon 17 in the TK domain (FLT3-TKD, also known as D835Y, and occurring in 5%-7% of AML patients) [8,16]. Both mutations contribute to constitutive activation of the FLT3 receptor [8]. It has been shown that the FLT3-ITD mutation has an inverse correlation with patient survival and can be used as an important poor prognostic factor to predict clinical outcomes in AML patients, especially those with normal karyotypes. However, data on the correlation between FLT3-TKD and AML disease outcome are highly limited [3,4,7,17].

The nucleophosmin gene encodes the NPM1 protein, which functions as a chaperone that shuttles between the nucleus and cytoplasm [3,5,7,8]. NPM1 regulates different intracellular processes such as transport of preribosomal particles, responses to stress stimuli, DNA repair, centromere duplications, and the activity and stability of tumor suppressor genes like p53 [3]. Mutation within exon 12 of the NPM1 gene, which is the most frequent mutation in AML patients (about 35% in all adult AML patients and 50%-60% of CN-AML cases), results in abnormal expression and localization of the protein within the cytoplasm [3]. The most common NPM1 mutation (type A mutation, occurring in 75%-80% of cases) is the insertion of the TCTG tetranucleotide at position 956-959 in exon 12, but other less common mutations in exon 12 have also been described [18,19]. There are various reports describing that NPM1 mutation is mostly associated with FLT3-ITD mutation and it has been shown that NPM1 can be considered a favorable prognostic marker in the absence of FLT3-ITD mutation [3,4,7,17].

Accordingly, in this study, FLT3 and NPM1 mutations were evaluated in adult Iranian patients with de novo CN-AML and its correlations with clinical and laboratory parameters were also assessed.

## MATERIALS AND METHODS

### Patient Selection

This study included 70 newly diagnosed adult patients with de novo AML who were referred to the Shiraz Namazi Hospital, affiliated to Shiraz University of Medical Sciences, from November 2014 to May 2016. All patients were recruited at the time of diagnosis prior to chemotherapy. AML was diagnosed using morphology, cytochemistry, and immunophenotyping. Clinical and laboratory data, including French-American-British (FAB) subclass, complete blood count, blast percentage, and hemoglobin (Hb) level, were also collected.

All patients received standard induction chemotherapy, which consisted of daunorubicin at 45 mg/m^2^ on days 1 to 3 and cytarabine at 100-200 mg/m^2^ on days 1 to 7, followed by high doses of a cytarabine-based consolidation phase (cytarabine at mg/m^2^ 3 every 12 h for 3 days, repeated for 2 to 3 cycles). This study was approved by the Ethics Committee of Shiraz University of Medical Sciences and written informed consent was obtained from all the participants.

### Cytogenetic Analysis

Karyotypes were analyzed by standard G-banding technique [20]. Chromosomal abnormalities were tested by reverse transcriptase polymerase chain reaction (PCR) for AML1-ETO and CBFB-MYH11. Among the 70 AML patients, 16 had abnormal karyotypes: one patient had inv (16) translocation, one had both t (8;21) and inv (16), 12 had t (15;17), and the remaining two patients had other translocations. The 54 patients who were negative for these chromosomal abnormalities were considered as having CN-AML.

### Sample Collection

Five milliliters of fresh peripheral blood and/or bone marrow samples was collected in ethylenediaminetetraacetic acid-containing tubes. DNA was extracted with a DNA extraction kit (GeNet Bio, Korea) and stored at -80 °C.

### Detection of the FLT3-ITD Mutation

For detection of the FLT3-ITD mutation, the JM domain between exons 11 and 12 was amplified using specific forward primer FLT.11F 5’-GCAATTTAGGTATGAAAGCCAGC 3’ and reverse primer FLT.12R 5’-CTTTCAGCATTTTGACGGCAACC-3’. The PCR reaction was performed in a total volume of 50 µL containing 200 ng of genomic DNA, 10X PCR buffer (100 mM Tris-HCl, pH 8.8, 500 mM KCl), 2 mM MgCl_2_, 200 µM dNTPs, 10 pM of each primer, and 1 U of Taq DNA polymerase. PCR conditions included initial denaturation at 95 °C for 5 min followed by 30 cycles of 94 °C for 30 s, 56 °C for 30 s, and 72 °C for 45 s with a final extension at 72 °C for 5 min. PCR reaction was conducted in a PCR T100 thermocycler (Applied Biosystems, USA). The 329-bp PCR products were run on 3% agarose gel stained with DNA SafeStain Dye and visualized under UV light. Samples with additional longer PCR products were identified as FLT3-ITD+. All mutant samples were verified by direct sequencing using the ABI Prism 3730XL DNA sequencing analyzer. The sequencing results were analyzed by Chromas software (version 2.4.3).

### Detecting of the FLT3-TKD Mutation

For detection of the FLT3-TKD mutation, the specific forward primer FLT.17F 5’-CCGCCAGGAACGTGCTTG-3’ and reverse primer FLT.17R 5’-GCAGCCTCACATTGCCCC-3’ were used. The PCR reaction was performed in a total volume of 15 µL with similar reagents as used for the FLT3-ITD mutation, except for the primers. PCR conditions were also the same, except for the annealing temperature, which was 65 °C for 30 s. The amplification reaction was conducted in a PCR T100 thermocycler (Applied Biosystems). The 119-bp PCR products were then digested with 2 U of EcoRV at 37 °C for 17 h, run on 3% agarose gel stained with DNA SafeStain Dye, and visualized under UV light. The presence of an undigested PCR product was an indication of a mutant sample.

### Detection of the NPM1 Mutation

Exon 12 of the NPM1 gene was amplified using specific primer NPM1-F 5’-TTAACTCTCTGGTGGTAGAATGAA-3’ and NPM1-R 5’-CAAGACTATTTGCCATTCCTAAC-3’. The PCR reaction was performed in a similar volume as was used for the FLT3-ITD mutation. PCR conditions included initial denaturation at 95 °C for 5 min followed by 30 cycles of 94 °C for 30 s, 57 °C for 60 s, and 72 °C for 75 s with final extension at 72 °C for 5 min. The PCR products were purified and directly sequenced with reverse primer NPM1-R2 5’-GGCATTTTGGACAACACA-3’ using the ABI Prism 3730XL DNA sequencing analyzer and analyzed by Chromas software (version 2.4.3).

### Statistical Analysis

The statistical analysis of data was done using SPSS 18 (SPSS Inc., USA). For comparison of qualitative data between wild-type and mutant patients, chi-square and Fisher exact tests were performed. Independent sample t-tests and Mann-Whitney U tests were used to compare quantitative data between wild-type and mutant patients. A p-value of less than 0.05 was considered statistically significant.

## RESULTS

This study included 70 newly diagnosed adult patients with de novo AML (49 males and 21 females, mean age: 47.73±18.64 years, minimum - maximum: 17-87 years). The demographic and laboratory data of all the patients are shown in Table 1.

### Screening for the Mutation of the FLT3 and NPM1 Genes in CN-AML

**Figure 1:**
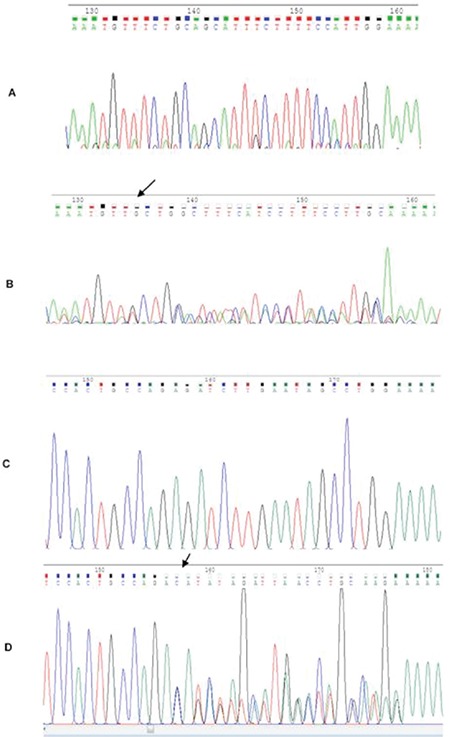
Sequencing results for FLT3-ITD and NPM1 mutation: A and B are representative of patients with wild-type and mutant FLT3-ITD gene, respectively. C and D are representative of patients with wild-type and mutant NPM1 gene, respectively. The arrows show the mutation site.

The chromatograms of FLT3-ITD and NPM1 sequencing are shown in Figure 1.

Of all 54 CN-AML patients, 14 (25.9%) had the FLT3-ITD mutation, while 40 (74.1%) had the normal FLT3 gene. In addition, of the 52 patients genotyped for FLT3-TKD mutation status, 3 (5.9%) were mutant and 48 (94.1%) were normal. One patient had both FLT3-ITD and FLT3-TKD mutations.

Of the 53 CN-AML patients genotyped for the NPM1 gene, 11 (20.8%) had NPM1 mutation and 42 (79.2%) had wild-type NPM1. From the 11 patients with mutant NPM1, 8 (72.8%) had type A, 1 (9.1%) had type C, and 1 (9.1%) had type D mutation. One patient (AML-20) had a unique mutation pattern that did not belong to a typical NPM1 mutation type. Of 11 patients with mutated NPM1, 5 (45.5%) were also positive for FLT3-ITD, while none had FLT3-TKD mutation. Thirty-three patients had the wild-type form of both the FLT3-ITD and NPM1 genes. There was no significant correlation between the presence of the FLT3-ITD mutation and NPM1 mutation in CN-AML patients (p>0.05).

### FLT3 and NPM1 Mutations and Different Clinical and Laboratory Parameters in CN-AML

The mean white blood cell (WBC) and platelet counts, serum Hb level, and percentage of blasts in the bone marrow were compared between mutant and wild-type groups of CN-AML patients (Table 2).

As shown in Table 2, there were no significant differences in mean WBC and platelet counts, serum Hb level, or percentage of blasts in the bone marrow between patients with wild-type and mutant FLT3-ITD and NPM1 genes. Moreover, the mean age of AML patients did not differ between wild-type and mutant patients for the FLT3-ITD and NPM1 mutations (p=0.287 and p=0.387, respectively). No significant differences were observed between male and female patients in cases of FLT3-ITD and NPM1 mutation frequency (p=0.450 and p=0.545, respectively).

### FLT3 and NPM1 Mutation in AML Patients with Different FAB Groups and Cytogenetic Aberrations

Of 70 de novo AML patients, 17 had FLT3-ITD, 3 had FLT3-TKD, and 12 had NPM1 mutations. The frequencies of these mutations in patients with different cytogenetic abnormalities are shown in Table 3. Although the frequency of FLT3-ITD, FLT3-TKD, and NPM1 mutation was higher in CN-AML patients in comparison with AML patients with cytogenetic aberrations, the differences were not statistically significant (p>0.05, data not shown).

Since the AML subtypes of some patients were not defined, AML patients were divided into M3 and non-M3 groups according to the FAB classification. As a result, 12 (17.1%) were M3 and 58 (82.9%) were non-M3. The FLT3 and NPM1 mutation status was analyzed in AML patients according to FAB groups. The results showed that there were no differences between the mutation status of the FLT3-ITD, FLT3-TKD, and NPM1 genes in the M3 and non-M3 FAB subtypes (Table 4). No mutation was observed in either FLT3-TKD or NPM1 genes in patients of the M3 FAB group. The FLT3-ITD mutation was more frequent in non-M3 patients compared to M3 patients (82.4% vs. 17.6%, respectively; Table 4).

## DISCUSSION

Genetic abnormalities are one of the most common features observed in AML patients, of which genetic variations of the FLT3, NPM1, DNMT3A, IDH1/2, and WT1 genes have been given more attention [3,7].

In the current study, we analyzed the frequency of FLT3 and NPM1 mutation in 54 adult de novo AML patients with normal karyotypes (CN-AML). The results showed that the frequency of FLT3-ITD, FLT3-TKD, and NPM1 mutations was 25.9%, 5.9%, and 20.8%, respectively. The most frequent NPM1 mutant type was the type A mutation. Our results are consistent with previous studies that described the FLT3-ITD mutation in 25%-35%, FLT3-TKD mutation in 7%-10%, and NPM1 in 50%-60% of CN-AML cases [7,21]. In a study of 39 CN-AML patients by Aly et al. [22], the frequency of FLT3-ITD was reported to be 15.4%, while Fröhling et al. [23] and Kainz et al. [24] found that the frequency of FLT3-ITD was 32% and 30% in CN-AML patients, respectively. In addition, Falini et al. [18] showed that the frequency of NPM1 mutation was 61.7%, while different mutation rates were reported by Zhang et al. [25] (14.3%), Döhner et al. [26] (48.3%), and Boissel et al. [27] (47%). The discrepancy in the frequency of FLT3-ITD, FLT3-TKD, and NPM1 mutation between our study and others may be due to different population groups as well as the number of cases in the abovementioned studies.

Consistent with previous reports, our results also demonstrated that the frequency of FLT3-ITD, FLT3-TKD, and NPM1 mutation was higher in CN-AML patients in comparison with AML patients with cytogenetic aberrations [3,7,28].

No mutation was detected in the FLT3-TKD or NPM1 gene in patients in the M3 FAB group. FLT3-ITD mutation was more frequent in non-M3 patients compared to M3 ones. Consistent with our results, Falini et al. [18], Thiede et al. [19], and Suzuki et al. [29] reported no NPM1 mutation in the M3 subtype. In addition, Verhaak et al. [30] reported a lower frequency of NPM1 mutation in M3 and M0 in comparison with other subgroups. Therefore, it seems that both FLT3 and NPM1 mutations are generally mostly seen in AML patients with normal cytogenetics.

Evaluation of the clinical characteristics of the patients revealed that there were no significant differences in mean WBC and platelet counts, serum Hb level, or bone marrow blast percentage between patients with wild-type and mutant FLT3-ITD and NPM1 genes. No difference was observed in the frequency of FLT3-ITD or NPM1 mutation regarding age or sex. Consistent with our findings, Dehbi et al. [31] reported no significant association between FLT3-ITD mutation and WBC and platelet counts or blast percentage. Bao et al. [32] also did not observe any differences in FLT3-ITD mutation frequency according to age or sex. However, higher WBC counts and increased blast percentages in FLT3-ITD-positive patients were reported by Fröhling et al. [23]. Moreover, Haferlach et al. [33] showed a strong association of bone marrow blast percentage with NPM1 and FLT3-ITD mutations. Gale et al. [28] and Döhner et al. [26] reported that a significant correlation existed between the presence of the FLT3-ITD mutation and the NPM1 mutation. However, there was no significant correlation between the concomitant mutation of both the FLT3-ITD and the NPM1 gene in our study, which might be due to the different sample sizes and also the type of AML (CN-AML in our study and unselected AML patients in the study by Gale et al. [28]).

It has been demonstrated that the FLT3-ITD mutation promotes constitutive activation of the FLT3 receptor, leading to ligand-independent cell stimulation and subsequent uncontrolled proliferation of leukemic blasts [3,8]. Mutation in exon 12 of NPM1 leads to aberrant cytoplasmic accumulation of the NPM1, which might contribute to leukemogenesis [21]. Association of the mutation in both of these genes with clinical outcome has been shown in various studies; NPM1 has been shown to be associated with good prognosis, especially in the absence of the FLT3-ITD mutation, while FLT3-ITD has been independently considered as a worse prognostic factor that significantly reduces patients’ survival [22,26,28,30,34,35].

According to our findings, the higher incidence of both the FLT3 and the NPM1 mutation in CN-AML patients underscores that both FLT3 and NPM1 can be used as candidate genetic markers for predicting the prognosis of CN-AML patients. In line with these genes, other known prognostic genetic markers like the DNMT3A and IDH genes should be considered, which are under further investigation by our group. Due to time limitations, it was not possible to follow our patients for a longer period of time in order to conduct survival analysis. However, further screening of patients for FLT3 and NPM1 mutations could be useful to verify the clinical significance of these genes for AML population prognosis, and especially for assessment of the presence of the remaining clones as minimal residual disease. In this regard, the value of increasing the number of patients in the studied population should be taken into account.

## CONCLUSION

In conclusion, given the high stability of NPM1 during the disease course, it can be used in combination with FLT3 as well as other known genetic markers to monitor Iranian CN-AML patients, especially for minimal residual disease detection.

## Figures and Tables

**Table 1:**
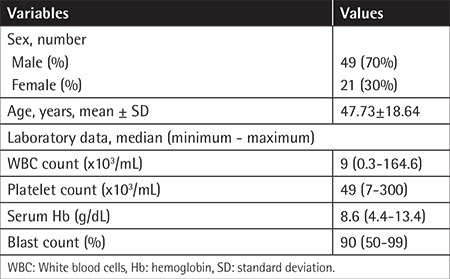
Demographic and laboratory data of acute myeloid leukemia patients.

**Table 2:**
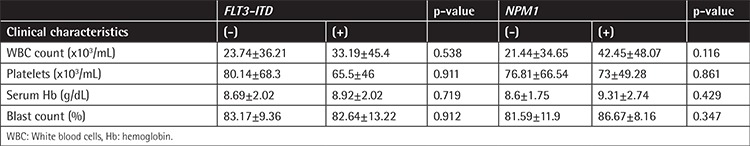
Comparison of baseline characteristics between wild-type and mutant groups.

**Table 3:**
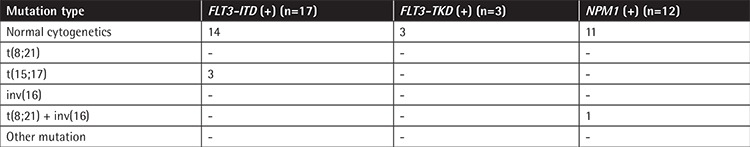
The frequency of FLT3-ITD, FLT3-TKD, and NPM1 mutations in acute myeloid leukemia patients with different cytogenetic statuses.

**Table 4:**
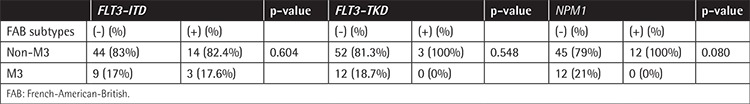
FLT3 and NPM1 mutation status in different French-American-British groups.
